# Drainage-Controlled Cellulose-Fiber Stabilization and Skeleton–Mastic Response of Polymer-Modified Stone Mastic Asphalt

**DOI:** 10.3390/polym18141769

**Published:** 2026-07-20

**Authors:** Ahmet Umutlu, Başak Varli Bingöl

**Affiliations:** 1 Republic of Turkey Ministry of Transport and Infrastructure, Ankara 06000, Turkey; ahmetumutlu@gmail.com; 2 Department of Civil Engineering, Çankiri Karatekin University, Çankırı 18000, Turkey

**Keywords:** SBS-modified bitumen, stone mastic asphalt, cellulose fiber, fiber-stabilized mastic, aggregate skeleton, SEM, FTIR

## Abstract

Stone mastic asphalt (SMA) derives its performance from the coupled action of a load-bearing coarse aggregate skeleton and a binder-rich mastic phase. This study evaluates polymer-modified SMA using an integrated drainage–skeleton–mastic framework that combines drainage-based cellulose fiber selection, controlled gradation variation, aggregate-type comparison, binder-content sensitivity analysis, pre-compaction laboratory conditioning, and FTIR–SEM–EDX characterization. A 50/70 penetration-grade bitumen modified with 4.5% SBS was used with basalt and limestone aggregates, limestone filler, and Viatop cellulose fiber. The fiber dosage was selected using the Schellenberg binder-drainage test, while a separate preliminary load–deformation series was used to examine the response sensitivity to higher fiber contents. Increasing fiber content from 0.30% to 0.35% reduced mean binder drainage from 0.27% to 0.18% and decreased the standard deviation from 0.020% to 0.006%, supporting 0.35% as a drainage-based design dosage rather than a mechanical optimum. Higher fiber contents increased the maximum recorded load within the fixed test window; however, these results were interpreted only as preliminary load–deformation sensitivity rather than as conventional Marshall stability or MQ responses. The binder-content series showed that lower- and upper-limit gradations followed different volumetric and Marshall response patterns; therefore, these results were interpreted as binder-content sensitivity rather than complete optimum binder content determination. Aggregate-type comparisons showed the mechanical advantage of basalt, while the non-replicated post-extraction gradation results were directionally consistent with greater skeleton preservation in basalt mixtures. FTIR, SEM, and EDX observations indicated that cellulose fiber acted mainly through physical mastic stabilization rather than chemical binder modification. Overall, the results demonstrate that SMA response is governed by the combined contribution of drainage-controlled fiber dosage, SBS-modified binder, aggregate skeleton configuration, and limestone-filler mastic integrity.

## 1. Introduction

Stone mastic asphalt (SMA) is a gap-graded hot-mix asphalt in which the main load-carrying function is provided by a coarse-aggregate skeleton with stone-on-stone contact. Unlike conventional dense-graded mixtures, the response of SMA cannot be adequately understood from binder content, air voids, or Marshall stability alone; it depends on the interaction between the load-bearing aggregate skeleton and the bituminous mastic phase filling the skeleton voids [1,2].

The high binder content of SMA improves cohesion and durability, but it also increases the risk of binder drainage during mixing, transport, and laying. Cellulose fibers are therefore commonly used as stabilizers. Their role is predominantly physical, as follows: they retain free binder, support the filler-bitumen mastic and help preserve the binder film within the aggregate skeleton [3,4,5]. In this sense, cellulose fiber is not merely an auxiliary additive, but a functional component of the SMA material architecture.

Recent studies confirm the central role of fiber-based binder stabilization in SMA and have increasingly focused on alternative and sustainable fibers. Valdés-Vidal et al. [6] developed a granular additive from waste-tyre textile fibers as a replacement for commercial cellulose and reported mechanical performance comparable to conventional cellulose-stabilized SMA, while Cabello-Suárez et al. [7] linked the chemical, morphological, and calorimetric properties of agro-industrial cellulose wastes directly to their drainage-control efficiency in the Schellenberg test. These works establish that the fiber governs SMA behavior primarily through binder retention and mastic stabilization, and that drainage performance, rather than mechanical reinforcement alone, is the governing selection criterion, which motivates the drainage-based fiber-selection procedure adopted in the present study. Polymer modification is equally central to modern SMA design: Albayati et al. [8], consistent with earlier SBS-modified SMA studies [9], showed that SBS- and polyethylene-modified mixtures exhibit improved rheological grade, moisture resistance, and rutting performance relative to unmodified binders, which supports the use of a constant 4.5% SBS-modified binder here so that the aggregate-skeleton and fiber–mastic variables can be isolated under a fixed, high-performance binder.

The filler is another critical component of the SMA mastic. Limestone filler, because of its mineral character and fine particle size, can affect binder retention, void filling, mastic consistency, and the distribution of binder within the mixture [10,11]. In the present study, limestone was therefore considered not only as an aggregate source in comparative mixtures, but also as the mineral component of the filler-mastic phase.

Aggregate lithology and mechanical resistance are also important because SMA performance is sensitive to the integrity of the coarse skeleton. A hard and abrasion-resistant aggregate such as basalt can better preserve the designed gradation under impact compaction, whereas a weaker or more flaky aggregate may experience breakage and produce a final post-compaction skeleton that differs from the target gradation [12].

The influence of aggregate lithology on SMA performance has been documented directly, as follows: Iskender [12] showed that basalt and basalt–limestone skeletons produce markedly different rutting responses, attributing the differences to the contrasting mechanical resistance and skeleton integrity of the two lithologies, and comparable basalt–limestone differences in SMA performance have been reported elsewhere [2]. This supports the treatment of aggregate type as a primary skeleton variable in the present study and the use of post-extraction gradation analysis to assess skeleton preservation.

More broadly, the functional performance of asphalt and alternative road-surface materials is governed by their void structure and drainage behavior across the full void spectrum. At the high-void end, permeability and drainage control the durability of alternative waste-based surface composites, as shown by Jin et al. [13] for high-air-void stamp-sand–acrylonitrile-styrene-acrylate plastic composites evaluated without asphalt binder, where permeability and aggregate loss scaled strongly with void level. Gap-graded SMA sits at the opposite, low-void end of this spectrum, where the high binder content promotes binder drain-down rather than permeability; this contrast clarifies why drainage control, not void reduction, is the governing design concern in SMA.

Despite this body of work, fiber dosage, binder modification, aggregate gradation, and aggregate lithology are typically studied in isolation, and the coupling between drainage-based fiber selection, skeleton preservation, and mastic stabilization within a single SBS-modified SMA system has received limited attention. The present study addresses this gap. Its objectives are as follows: (i) to select the cellulose-fiber dosage from binder-drainage performance and then examine its load–deformation response; (ii) to evaluate the sensitivity of the volumetric and Marshall response to aggregate gradation and binder content for a constant SBS-modified binder–fiber system; (iii) to compare basalt and limestone as skeleton-forming aggregates, including post-compaction skeleton preservation; and (iv) to interpret the fiber–bitumen interaction mechanistically through FTIR, SEM, and EDX analysis. These objectives are pursued within an integrated skeleton–mastic evaluation framework supported by ANOVA, MCDM, and PCA.

**Figure 1:**
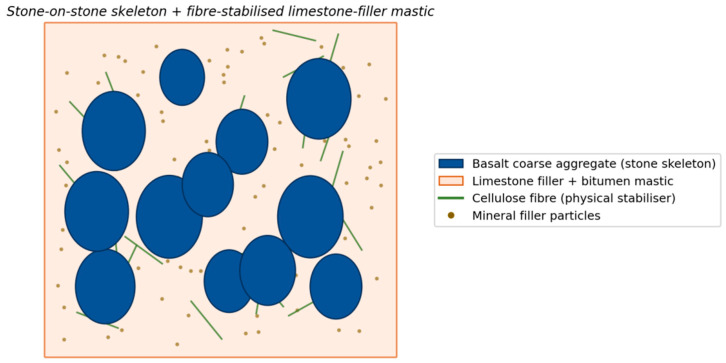
Conceptual representation of the stone-on-stone basalt skeleton and fiber-stabilized limestone-filler mastic in SMA.

The novelty of this study lies in the integrated evaluation of cellulose-fiber-stabilized, SBS-modified SMA as a coupled skeleton–mastic system rather than in the isolated use of cellulose fiber alone. Unlike studies that examine fiber dosage, aggregate gradation, or binder modification separately, this work combines a drainage-based fiber selection procedure, a constant 4.5% SBS–modified binder system, controlled SMA gradation variations, basalt–limestone aggregate comparison, post-extraction gradation analysis, and FTIR–SEM–EDX characterization within a single experimental framework. The selected 0.35% cellulose-fiber content is established from Schellenberg drainage performance and then examined through a preliminary load–deformation (fiber-content sensitivity) series, while higher fiber contents are treated as sensitivity levels rather than design candidates. In this way, the study links binder-drainage control, aggregate skeleton preservation, mastic stabilization, and volumetric–mechanical response (Figure 1). A further contribution is the use of complementary statistical and multi-criteria tools, including ANOVA, TOPSIS, Derringer desirability, and PCA, to compare the relative proximity of the tested trial mixtures to the selected response targets and to identify the governing response patterns of the SMA system. Therefore, the contribution of the study is not simply the addition of cellulose fiber, but the development of an experimentally supported evaluation framework for assessing how fiber dosage, SBS-modified binder, aggregate skeleton configuration, and mastic microstructure jointly control SMA performance [9,14].

## 2. Materials and Methods

### 2.1. Experimental Design

**Figure 2:**
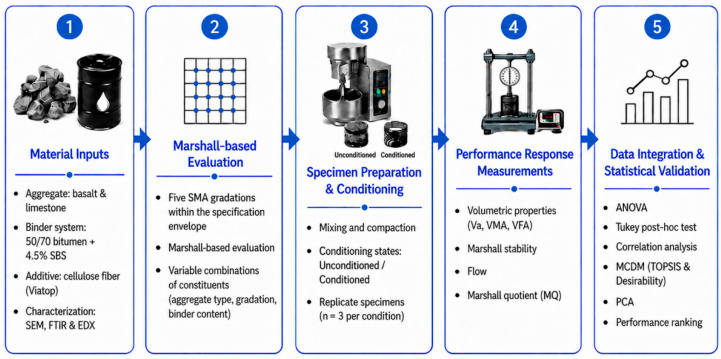
Experimental workflow adopted for the integrated SMA evaluation.

This study was designed to evaluate stone mastic asphalt (SMA) as a compacted pavement geomaterial by systematically investigating the combined effects of aggregate type, gradation-controlled skeleton formation, and pre-compaction laboratory conditioning on its volumetric and mechanical response. The overall experimental program is summarized in Figure 2 and comprised five stages. The first stage focused on material inputs, where the constituent materials were selected and characterized, including basalt and limestone aggregates, a polymer-modified binder (50/70 penetration-grade bitumen modified with 4.5% SBS), and cellulose fiber as a stabilizing additive. The fiber–mastic system was further characterized by FTIR, SEM, and EDX analyses. The second stage established the mixture design matrix by defining six SMA gradations within the SMA Type-1 specification. In the third stage, mixtures were mixed and compacted into Marshall specimens under both unconditioned and laboratory-conditioned states, with three replicate specimens prepared for each condition to provide statistical reliability. The fourth stage involved determining volumetric properties together with Marshall stability, flow, and Marshall quotient (MQ) for every mixture and conditioning state. Finally, the experimental results were integrated and evaluated using descriptive comparison, correlation analysis, regression analysis, ANOVA, multi-criteria decision-making methods, and principal component analysis.

Although the original experimental program contained several gradation and aggregate combinations, the present manuscript gives priority to the data that directly support the material-system framework. Dense-graded reference mixtures and mixtures containing a different basalt source were not emphasized in the main interpretation because they introduce additional confounding variables and do not represent the same SMA skeleton–mastic system.

### 2.2. Materials

Basalt aggregate was selected as the primary material forming the load-bearing coarse skeleton. Limestone was evaluated in two roles, as follows: as an aggregate in comparative mixtures and, more importantly, as the mineral filler representing the filler-mastic phase. Both the basalt and limestone aggregates were sourced from a quarry in Ankara, Turkey. Aggregates were washed to reduce uncontrolled fines, oven dried, and separated into fractions before mixture preparation. The physical and mechanical properties of the aggregates were determined according to the relevant TS EN and ASTM standards [15,16,17,18], and the results are presented in Table 1 and Table 2. The aggregate characterization included bulk and apparent specific gravity, water absorption, Los Angeles abrasion loss, Micro-Deval abrasion loss, methylene blue value, and flakiness index. In general, the basalt aggregate exhibited lower abrasion loss, lower methylene blue value, and lower flakiness index than the limestone aggregate, indicating a mechanically stronger and cleaner aggregate structure. These differences were considered important for interpreting the variations in structural response observed among the mixtures. Although basalt is generally associated with a relatively dense mineral structure, the basalt aggregate used in this study exhibited higher water absorption than the limestone aggregate (1.61% and 1.73% for the coarse and fine basalt fractions, respectively, compared with 0.55% and 0.41% for limestone; Table 1). This difference suggests that the tested basalt aggregate had a larger accessible pore volume, potentially associated with surface-connected pores, vesicles, or microcracks [2,12]. In asphalt mixtures, greater aggregate absorption may reduce the proportion of binder remaining available within the effective mastic phase and may influence binder-film development at the aggregate surface. However, binder absorption, effective binder content, and binder-film thickness were not measured directly in the present study. Therefore, the possible implications for adhesion, moisture susceptibility, and durability are considered only as qualitative factors and are not used as direct explanations of the observed mechanical response.

A 50/70 penetration-grade bitumen modified with 4.5% SBS was used as the binder throughout the experimental program. The bitumen was obtained from the asphalt plant and bitumen depot, Ankara, Turkey. The conventional properties of the modified binder, including penetration, softening point, ductility, elastic recovery, mass loss after RTFOT, and post-RTFOT penetration and softening point, are summarized in Table 3. These results were used to characterize the consistency, temperature susceptibility, elastic response, and short-term ageing behavior of the binder. A performance grade was not assigned because the rheological and low-temperature tests required for complete PG classification were not conducted in the present study.

**Figure 3:**
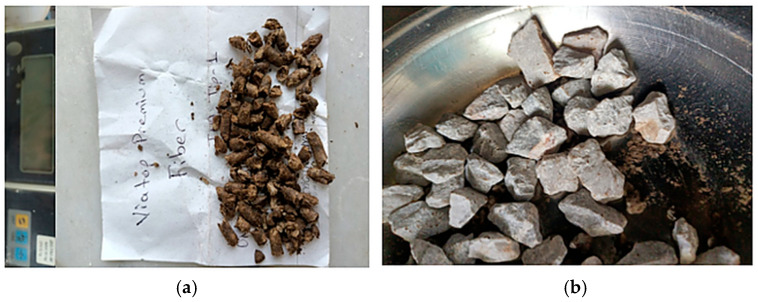
(**a**) Viatop premium fiber and (**b**) fragmented fiber structure.

Viatop Premium cellulose fiber (J. Rettenmaier & Söhne GmbH + Co. KG, Rosenberg, Germany) was used to control binder drainage (Figure 3). The fiber content was evaluated by the Schellenberg drainage test at 6.5% binder content [19,20]. In the main SMA mixtures, 0.35% fiber by mixture mass was used as a fixed stabilizing component. This selection was not intended to represent the fiber content giving the maximum recorded load; rather, it was adopted because it provided a safer drainage margin than the lower dosage evaluated.

The Viatop Premium additive is a pelletized cellulose-fiber granulate used as a binder-stabilizing agent for SMA and gap-graded mixtures. Its principal supplier-reported physical properties are summarized in Table 4. To characterize the fiber morphology quantitatively, individual fiber diameters were measured from the SEM micrographs (Section 3.4) across six fields at 250–500× magnification using automated image analysis (*n* = 24,252 fibers). The fibers are fine, filamentary, and broadly distributed in diameter, with a mean of 9.97 μm and a median of 6.50 μm (Table 5); ninety percent of the measured diameters fell between 1.8 and 19.6 μm. This fine, high-aspect-ratio, network-forming morphology is consistent with the binder-retention (drainage-control) function of the fiber discussed in Section 3.1 and Section 3.4.

### 2.3. Trial SMA Gradations and Binder-Content Sensitivity Design

**Figure 4:**
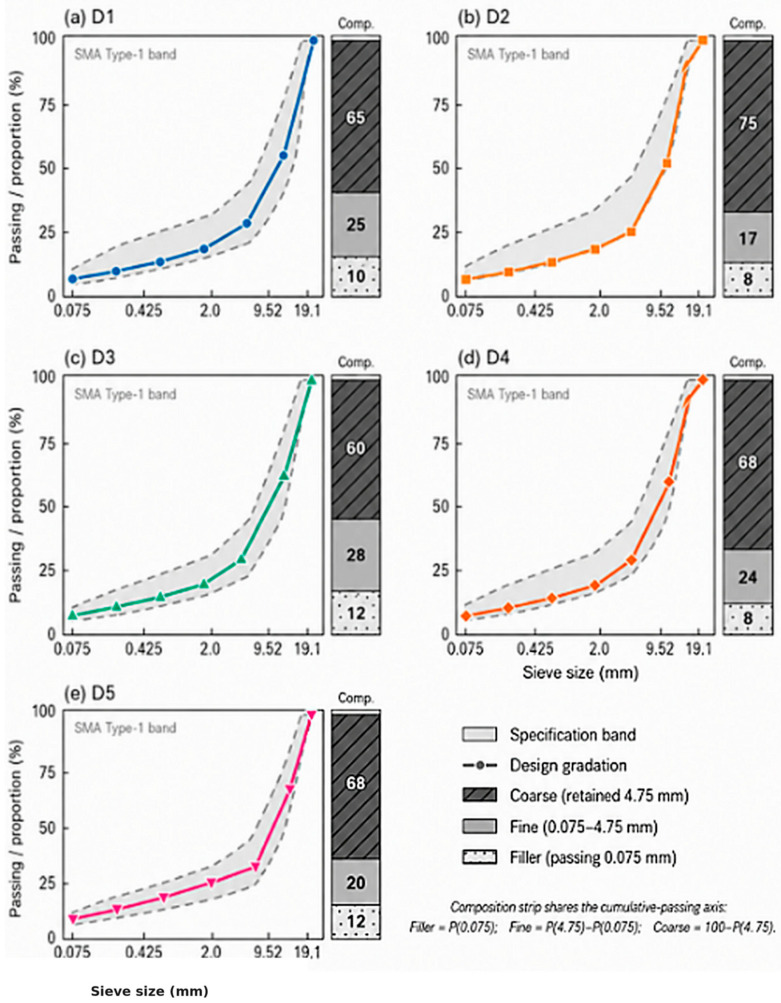
Aggregate gradation of mixtures on a logarithmic sieve-size axis. Note: In each panel (**a**–**e**), the colored curve represents the design gradation of the corresponding mixture; the grey shaded area denotes the SMA Type-1 specification band, the dashed lines indicate its upper and lower limits, and the composition strip on the right shows the coarse, fine, and filler proportions (%).

The experimental gradation matrix was established as a controlled trial series rather than as a set of fully optimized production-ready SMA mix designs. The purpose was to define how different aggregate skeleton configurations respond under the same binder–modifier–fiber system [21]. For that, six SMA-type gradations were selected within the SMA Type-1 specification envelope to represent different coarse aggregate, fine aggregate, and filler proportions (Figure 4). The gradation series covered coarse aggregate contents between 60% and 75%, fine aggregate contents between 17% and 28%, and filler contents between 8% and 12%. This enabled the influence of skeleton configuration on volumetric properties, Marshall stability, flow, and Marshall quotient to be compared under controlled material conditions. Structure of experimental series is shown in Table 6. Accordingly, mixtures that did not meet the target volumetric requirements were not interpreted as acceptable SMA design alternatives, but as diagnostic cases showing the sensitivity of the skeleton-mastic system to aggregate gradation and aggregate type. The binder-content series was not intended to determine a final optimum binder content for each mixture. Instead, it was used to evaluate the binder-content sensitivity of selected trial gradations under a constant SBS-modified binder and cellulose-fiber system. Therefore, the binder-content results should be interpreted as a comparative response analysis, not as a complete Marshall mix-design optimization.

### 2.4. Specimen Preparation and Testing

Aggregate fractions and the filler material were weighed in accordance with the target gradation, dried before mixing, and brought to the mixing temperature. The SBS-modified bitumen was heated to the appropriate mixing temperature, and the cellulose fiber was incorporated so as to be homogeneously distributed within the bitumen–filler–aggregate system. The binder was modified with 4.5% SBS to improve the mechanical characteristics of the binder phase, at one level according to recent studies [9,14]. Mixing was carried out to ensure the dispersion of the fiber within the mastic phase and the coating of the aggregate surfaces by the bitumen.

Twelve cylindrical specimens were assigned to four fiber-content groups with three replicate specimens per group, as follows: 0% (fiber-free control), 0.35%, 1%, and 2% fiber by total mixture mass. The load–deformation response was recorded up to a fixed ram travel of 10 mm. Unlike the main experimental series, the preliminary fiber-content specimens did not exhibit a distinct peak followed by post-peak softening within the applied test stroke. Therefore, the maximum recorded load represents the highest load reached within the 10 mm test window rather than a standard Marshall stability value corresponding to a clearly identified failure peak. Similarly, the deformation at this maximum recorded load should not be interpreted as standard Marshall flow, and the resulting load-to-deformation ratio should not be reported as a conventional Marshall quotient. Accordingly, the fiber-content series was used only as a preliminary within-series assessment of load–deformation sensitivity. Its results were not compared directly with the standard Marshall stability, flow, or MQ values obtained for the main gradation, aggregate-type, and conditioning series.

Marshall specimens were prepared in standard Marshall molds and compacted by applying 75 blows with the Marshall hammer (Utest Material Testing Equipment, Ankara, Turkey) to each face [22]. To improve repeatability and statistical robustness, three replicate specimens were produced and tested for each mixture condition. Accordingly, the adopted design framework provided methodological compatibility with Marshall-based SMA evaluation approaches used in Turkish practice as well as ASTM/AASHTO/EN-oriented pavement engineering applications. To evaluate the effect of pre-compaction thermal exposure, the loose SMA mixtures assigned to the conditioned state were subjected to laboratory oven (Utest Material Testing Equipment, Ankara, Turkey) conditioning before compaction. In this procedure, the mixtures were held at the compaction temperature for 60 min and then compacted using the standard Marshall procedure [23]. The unconditioned mixtures were compacted immediately after mixing. This conditioning procedure was used to represent the additional thermal exposure that may occur during plant production, transport, and paving; however, it should be interpreted as a controlled laboratory pre-compaction conditioning protocol rather than as a standardized aging procedure. The prepared Marshall specimens were evaluated in terms of volumetric properties and Marshall-based mechanical responses in order to interpret the relationship between the coarse-aggregate skeleton and the mastic phase. For each mixture condition, Marshall stability and flow tests were applied using a Marshall stability testing apparatus (Utest Material Testing Equipment, Ankara, Turkey). In addition, the Marshall quotient was calculated as the ratio of the stability value to the flow value and used as a comparative indicator of the stiffness–deformation balance of the mixture.

### 2.5. Statistical Evaluation

All statistical analyses (descriptive statistics, ANOVA, correlation and regression analysis, TOPSIS, the Derringer desirability function, and principal component analysis) were performed in Python (version 3.11). The experimental results were first evaluated using descriptive statistics, including mean values and standard deviations. Where the experimental design allowed direct comparison among groups, analysis of variance (ANOVA) was used to examine whether the observed differences were statistically meaningful. Post hoc comparisons were applied only when a significant overall effect was detected. Because each mixture condition was represented by a limited number of replicate specimens, the statistical results were interpreted as supporting evidence rather than as the sole basis for performance ranking. Therefore, the discussion prioritized mean trends, effect direction, engineering consistency, and agreement with the observed mixture structure. Correlation and regression analyses were used only as exploratory tools to support the interpretation of relationships among volumetric and Marshall parameters.

For the binder-content series, a two-way analysis of variance (gradation × binder content) including the interaction term was additionally used to test whether the binder-content response differed between the lower- and upper-limit gradations; effect sizes are reported as partial eta-squared (η^2^p).

To compare the aggregate-type mixtures across all Marshall responses simultaneously, two complementary multi-criteria decision-making (MCDM) methods were applied, as follows: the Technique for Order of Preference by Similarity to Ideal Solution (TOPSIS) and the Derringer desirability function [24,25]. Marshall stability, VMA, and Marshall quotient were treated as larger-the-better criteria, whereas air voids, VFA and flow were treated as target-the-best criteria with targets of 4%, 70%, and 3 mm, respectively. For TOPSIS, the decision matrix was vector-normalized and weighted, and the closeness coefficient (C_i_) of each mixture to the positive and negative ideal solutions was computed; three weighting schemes (equal, base, and stability-emphasized) were examined as a sensitivity check. For the desirability analysis, each response was scaled to a 0–1 desirability and combined as a weighted geometric mean. The criteria weights and target values were analyst-defined and should be aligned with the governing mix-design specification; the resulting rankings are therefore conditional on these settings. The MCDM analysis was used only as a screening tool to compare the relative conformity of the tested trial mixtures to the selected volumetric and Marshall criteria. It was not used to declare a final acceptable SMA mix design. Mixtures with poor volumetric conformity were retained in the analysis only to demonstrate how unsuitable skeleton–mastic configurations are penalized by the evaluation framework.

The Marshall quotient (MQ), defined as the ratio of Marshall stability to flow, is used in this study as an empirical stiffness surrogate. It should be noted that MQ has well-recognized limitations and does not correlate strongly with actual field rutting performance; it is therefore interpreted here only as a comparative, relative index among the tested mixtures, and not as a predictor of in-service rutting resistance. A reliable rutting assessment would require performance-based testing such as wheel-tracking or dynamic modulus measurement.

Finally, principal component analysis (PCA) was applied to the standardized Marshall responses of all specimens to characterize the multivariate structure of the data set [26]. Components were extracted by singular value decomposition, and the results are interpreted through the explained variance and the component loadings.

### 2.6. Chemical and Microstructural Characterization

To complement the mechanical evaluation, the cellulose fiber and its interaction with the bituminous phase were characterized by Fourier-transform infrared (FTIR) spectroscopy and scanning electron microscopy (SEM) [14]. FTIR spectra were recorded for the neat 50/70 penetration-grade bitumen and the 50/70 bitumen–cellulose fiber combination over the 4000–400 cm^−1^ range using a Bruker Tensor II spectrometer (Bruker Optik GmbH, Ettlingen, Germany) in attenuated total reflectance (ATR) mode. The spectra were exported directly from the instrument software (Bruker OPUS, version 7.5.18; Bruker Optik GmbH, Ettlingen, Germany) without smoothing or baseline manipulation. Because band intensities in ATR measurements depend on the sample–crystal contact, the two spectra were compared on the basis of band position rather than absolute intensity, and characteristic cellulose band positions were taken from the literature. The spectra were used to identify the characteristic functional groups of each constituent and to determine whether incorporation of the fiber into the bitumen produced new absorption bands or band shifts indicative of chemical interaction. The dispersion and surface morphology of the cellulose fiber within the bituminous mastic, together with the fracture-surface microstructure of the binder phase, were examined using a field-emission scanning electron microscope (FE-SEM; ZEISS Sigma 300 VP; Carl Zeiss Microscopy GmbH, Oberkochen, Germany) operated at an accelerating voltage of 20 kV with a secondary-electron (SE2) detector under high-vacuum mode. Micrographs were acquired at magnifications between 250× and 2000× to capture both the overall fiber distribution and the fine surface texture of the mastic. The elemental composition of a representative fiber-containing region was additionally determined by energy-dispersive X-ray spectroscopy (EDX; Bruker Nano GmbH, Berlin, Germany) coupled to the microscope, using a full-area scan at 20 kV. In addition, the SEM micrographs were processed by a Python (version 3.11) (scikit-image) image-analysis routine to quantify the apparent cellulose-fiber diameter.

## 3. Results and Discussion

### 3.1. Drainage-Based Fiber Selection and Marshall Response

The design fiber dosage was selected primarily on the basis of the Schellenberg binder-drainage test. Two fiber contents within the conventional dosage range for stone mastic asphalt mixtures, 0.30% and 0.35% by total mixture mass, were evaluated at a constant binder content of 6.5%. Both dosages produced mean drainage values below the commonly applied 0.3% limit, as follows: 0.27% at 0.30% fiber and 0.18% at 0.35% fiber (Table 7). However, the drainage variability at 0.30% fiber was greater (SD = 0.020%), and its upper variability band reached the specification threshold (mean + 2SD ≈ 0.31%). In contrast, the 0.35% dosage produced a lower mean drainage value and substantially less variability (SD = 0.006%; mean + 2SD ≈ 0.19%). Therefore, 0.35% fiber was selected not because 0.30% was categorically non-compliant, but because it provided a wider and more consistent margin against binder drain-down. This dosage should accordingly be regarded as a drainage-controlled design selection rather than a mechanically optimized fiber content.

**Figure 5:**
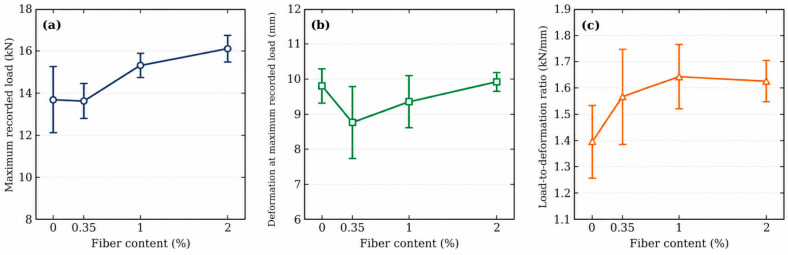
Effect of fiber content on (**a**) maximum recorded load, (**b**) deformation at the maximum recorded load, and (**c**) load-to-deformation ratio.

The preliminary load–deformation responses obtained at 0%, 0.35%, 1%, and 2% fiber are presented in Figure 5. The maximum recorded load remained nearly unchanged between the fiber-free control and the selected 0.35% dosage, with mean values of 13.70 and 13.64 kN, respectively. It subsequently increased to 15.33 kN at 1% fiber and 16.13 kN at 2% fiber. The deformation corresponding to the maximum recorded load decreased from 9.81 mm for the control to 8.77 mm at 0.35% fiber and then increased progressively, reaching 9.92 mm at 2% fiber. Consequently, the load-to-deformation ratio increased at 0.35% fiber, reached its highest mean value of 1.644 kN/mm at 1% fiber, and then decreased slightly to 1.626 kN/mm at 2% fiber.

These results indicate that increasing the fiber content altered the load–deformation response within the preliminary test series. In particular, the higher dosages were associated with greater loads within the applied test window, which may reflect increased physical restraint of the binder-rich mastic through fiber entanglement and binder retention. However, the load–deformation curves did not exhibit a distinct failure peak followed by post-peak softening within the fixed 10 mm ram travel. The reported maximum loads therefore represent the highest loads reached within the test window rather than standard Marshall stability values corresponding to a clearly identified failure peak. For the same reason, the associated deformation and load-to-deformation ratio should not be interpreted as conventional Marshall flow and Marshall quotient, respectively. These parameters are used only to describe relative trends within the fiber-content series and are not directly comparable with the standard Marshall results obtained for the main experimental series.

Accordingly, the preliminary mechanical series does not establish a mechanical optimum or independently confirm compliance of the evaluated fiber dosages with Marshall performance criteria. The 1% and 2% fiber contents are retained only as higher-dosage sensitivity levels illustrating how the recorded load–deformation response changed as fiber content increased. The selection of 0.35% remains governed by its lower and more consistent Schellenberg drainage result, its position within the conventional SMA dosage range, and the absence of a marked reduction in the maximum recorded load relative to the fiber-free control within this preliminary series. This interpretation is consistent with the FTIR and SEM observations in Section 3.4, which indicate that the cellulose fiber acts predominantly through physical binder retention and mastic stabilization rather than chemical modification of the binder.

### 3.2. Gradation-Controlled Skeleton Response and Binder-Content Sensitivity

**Figure 6:**
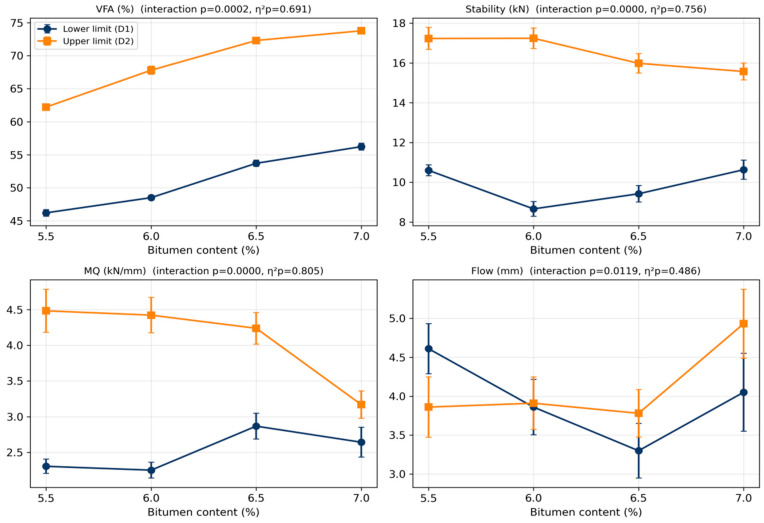
Interaction plots for the responses showing a significant interaction.

Figure 6 shows that the lower- and upper-limit gradations produced clearly different volumetric and Marshall responses. The ANOVA results supported this observation, indicating that gradation had a statistically significant effect on the main response parameters, including air voids, VMA, VFA, Marshall stability, and MQ. Binder content also influenced several responses; however, its effect was not uniform for the two gradations. In particular, the gradation × binder-content interaction indicated that the lower- and upper-limit gradations did not follow the same binder-response pattern. This finding is important from a mixture-design perspective because it shows that SMA behavior cannot be interpreted only through binder content; the aggregate skeleton configuration also controls how the mixture responds to binder variation.

**Figure 7:**
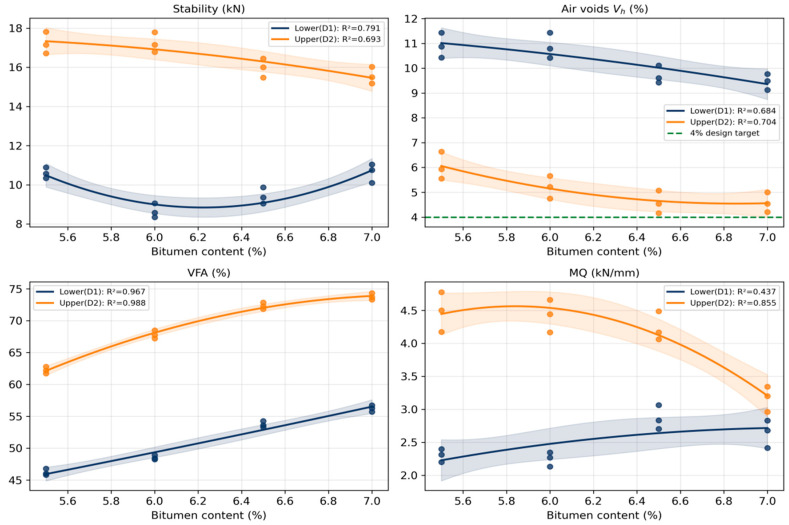
Fitted second-order curves with 95% confidence bands; the dashed line marks the 4% air-void design target.

The fitted curves were used to visualize the binder-content response of the two selected gradations. Although some responses, particularly VFA, were well represented by second-order trends, the regression models were not used to extrapolate a final optimum binder content. The main purpose of the modeling was to show that the two gradation structures responded differently to binder-content variation. Since the fitted air-void curves did not reach the conventional 4% target within the investigated binder range, the results were interpreted as binder-content sensitivity rather than as a complete OBC determination (Figure 7).

**Figure 8:**
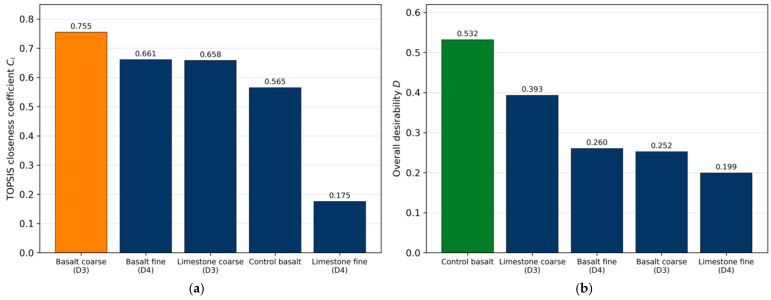
Multi-criteria ranking of the aggregate-type mixtures: (**a**) TOPSIS closeness coefficient under three weighting schemes and (**b**) Derringer overall desirability. (In each panel, the highlighted bar (orange in (**a**); green in (**b**)) denotes the top-ranked mixture).

Figure 8 presents the multi-criteria ranking of the aggregate-type mixtures. TOPSIS ranked basalt-coarse (D3) highest (C_i_ = 0.755) because its air voids (4.51%) and VFA (73.5%) were closest to the design targets, whereas the desirability function favored control basalt (D = 0.532) owing to its highest stability (13.86 kN); the divergence reflects target conformance versus mechanical capacity rather than an inconsistency. Both methods nonetheless agreed that the basalt mixtures outperformed the limestone mixtures and that limestone-fine (D4) was clearly the poorest alternative (C_i_ = 0.175; D = 0.199), consistent with its excessive air voids (10.24%) and lowest VFA (52.4%). As the rankings depend on the analyst-defined weights, targets, and limited replicates (*n* = 3), the closely ranked alternatives should not be regarded as meaningfully different.

The TOPSIS and desirability rankings are used here as a relative, multi-response comparison of the tested mixtures under a transparent, analyst-defined preference scheme; they are not intended to identify a design-optimal or specification-compliant mixture. Because none of the mixtures lies at the 4% air-void reference, the air-void criterion functions as a distance-to-reference measure rather than a compliance measure, so the rankings indicate which tested alternative is comparatively closest to the stated preference, not which alternative is acceptable in absolute terms. To probe the influence of the weight choice, the ranking was evaluated under three weighting schemes (Figure 8a) and is reported as a sensitivity check rather than as a definitive ordering. The independent contribution of flow was then examined directly by recomputing the ranking with flow removed from the criterion set, using the same decision matrix, normalization, and target definitions. Under equal criterion weights, the TOPSIS ordering of the aggregate-type mixtures remained unchanged (Spearman ρ = 1.00, Kendall τ = 1.00), with basalt-coarse ranked highest and limestone-fine lowest in both cases, and the top-ranked alternatives of the desirability analysis were likewise preserved; the same invariance was obtained under an alternative stability-emphasized weighting scheme (ρ = 1.00). These results confirm that flow contributes negligible independent discriminating information to the multi-criteria ranking, consistent with its short loading vector in the PC1–PC2 biplot. Flow is nevertheless retained among the reported criteria because it is a standard, specification-relevant Marshall response and is required to compute the Marshall quotient (Supplementary Materials, Tables S1 and S2). The two gradations were selected to bracket the skeleton response rather than to force a single volumetric target, as follows: within the tested 5.5–7.0% binder range, the upper-limit gradation approached the 4% air-void reference (≈4.5%), whereas the lower-limit gradation remained deliberately void-rich (≈10%), and no optimum binder content was fitted or extrapolated from these trial series.

**Figure 9:**
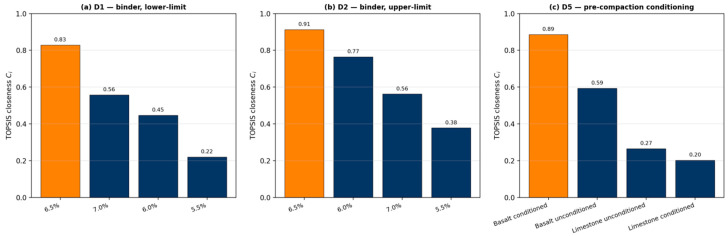
Within-design TOPSIS ranking (base weights; orange = best alternative) for (**a**) the lower-limit binder series (D1), (**b**) the upper-limit binder series (D2), and (**c**) the pre-compaction conditioning design (D5).

The same TOPSIS procedure was applied within the remaining designs (Figure 9). For both gradations, the binder-content ranking ranked 6.5% highest among the tested binder levels (closeness coefficient 0.83 for the lower-limit and 0.91 for the upper-limit gradation), consistent with the binder-content sensitivity discussed above. For the conditioning design (detailed in Section 3.3), the basalt mixtures ranked above the limestone mixtures. This is a relative ranking among the tested levels and is not presented as a production optimum.

**Figure 10:**
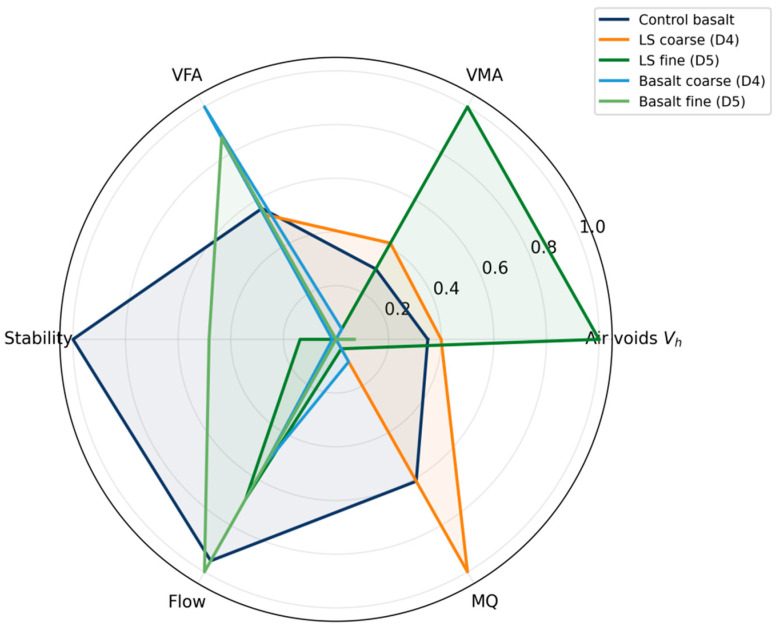
Normalized Marshall profiles of the five mixtures.

The normalized radar profiles (Figure 10) summarize the relative performance of the five mixtures across the six Marshall parameters, each scaled between the minimum and maximum values of the set. The basalt-coarse and basalt-fine mixtures exhibited the most favorable volumetric signatures, combining the lowest air voids with the highest VFA, indicating the densest and best-filled skeleton, whereas the control basalt mixture was distinguished by the largest stability lobe, confirming its superior mechanical capacity. The limestone-coarse mixture showed a characteristic peak in Marshall quotient together with the lowest flow, reflecting a stiffer but more brittle response. In contrast, the limestone-fine mixture extended towards the air-void and VMA axes while collapsing on the VFA axis, an unfavorable profile denoting an over-voided, under-filled structure that is consistent with its lowest overall ranking. It should be noted that, because the axes are min–max normalized, high values on the air-void and VMA axes are not desirable; the radar therefore corroborates the multi-criteria analysis in identifying the basalt mixtures as volumetrically superior and the limestone-fine mixture as the least suitable.

**Figure 11:**
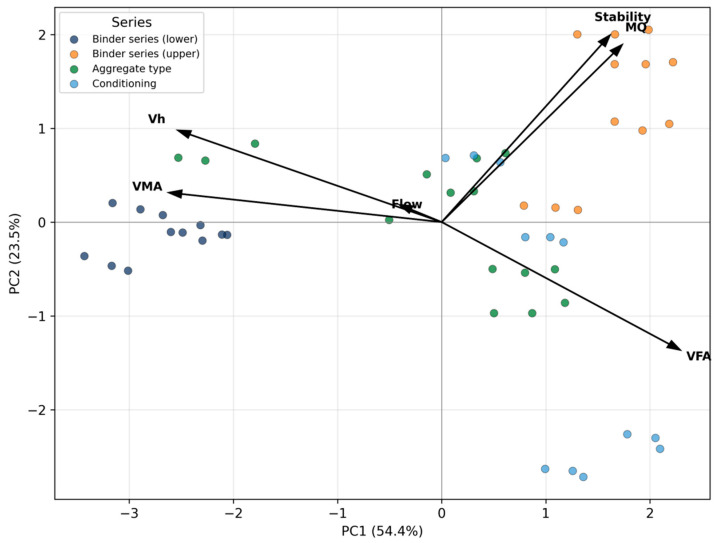
Principal component analysis biplot of the Marshall parameters; arrows denote parameter loadings and points denote specimens grouped by experimental series.

The principal component analysis biplot (Figure 11) captured 77.9% of the total variance in the first two components (PC1 = 54.4%, PC2 = 23.5%). PC1 represents a densification–strength axis, contrasting the void-related parameters (air voids and VMA, loading negatively to the left) with VFA, stability, and Marshall quotient (loading positively to the right), while flow exhibited a short loading vector close to the origin, indicating that it is only weakly represented by these two components and adds little independent discriminating information among the present mixtures. The four experimental series occupied distinct regions of the component space, as follows: the lower-limit binder series clustered on the high-void, low-strength (left) side, the upper-limit binder series grouped in the high-stability and high-MQ (upper-right) region, and the aggregate-type and conditioning specimens occupied the intermediate to lower-right area associated with higher VFA. This clear separation corroborates the analysis of variance in identifying gradation and aggregate configuration as the dominant sources of variation, and confirms that the volumetric and mechanical responses are strongly interrelated and can be summarized by two underlying dimensions.

### 3.3. Apparent Response to Pre-Compaction Conditioning

**Figure 12:**
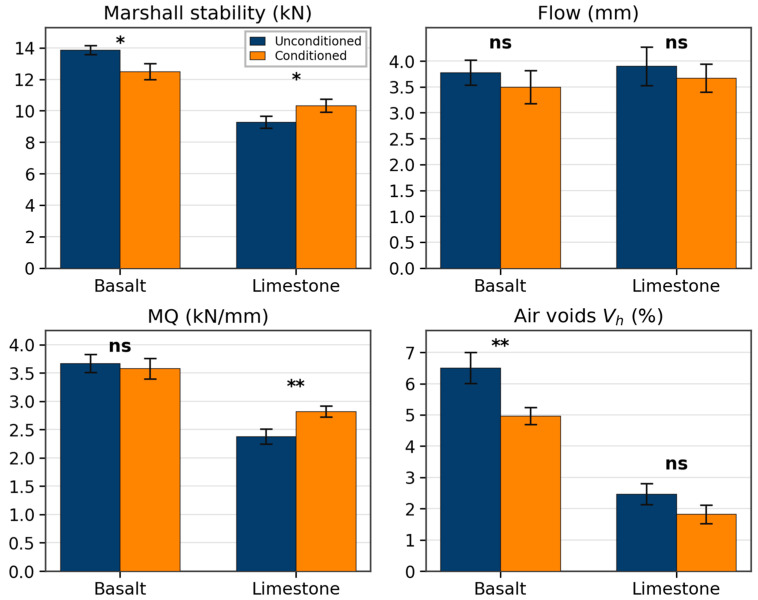
Effect of pre-compaction conditioning on the Marshall response of the basalt and limestone mixtures (mean ± SD; *t*-test markers: * *p* < 0.05, ** *p* < 0.01, ns = not significant).

The effect of pre-compaction laboratory conditioning on the Marshall response of the basalt and limestone mixtures is summarized in Table 8 and Figure 12. Conditioning reduced the mean air-void content of both mixtures, whereas the Marshall stability responses differed between the two aggregate systems. Basalt stability decreased from 13.86 to 12.51 kN, while limestone stability increased from 9.27 to 10.33 kN.

Because the conditioned and unconditioned specimens were not matched at a common air-void level, the effects of pre-compaction thermal holding and the accompanying change in densification cannot be separated quantitatively. The air-void content decreased from 6.51% to 4.97% in the basalt mixture and from 2.47% to 1.82% in the limestone mixture. These changes occurred from substantially different initial air-void levels and therefore should not be interpreted as equivalent densification histories solely on the basis of their relative percentage reductions. Despite the reduction in air voids in both mixtures, Marshall stability decreased for basalt and increased for limestone. The opposite trends suggest that aggregate type may influence the combined conditioning–densification response; however, the present results do not establish the mechanism responsible for this difference. Testing at controlled and comparable air-void levels is required to isolate the effect of pre-compaction thermal exposure from that of densification.

### 3.4. Aggregate Breakage and Fiber-Stabilized Mastic Mechanism

**Figure 13:**
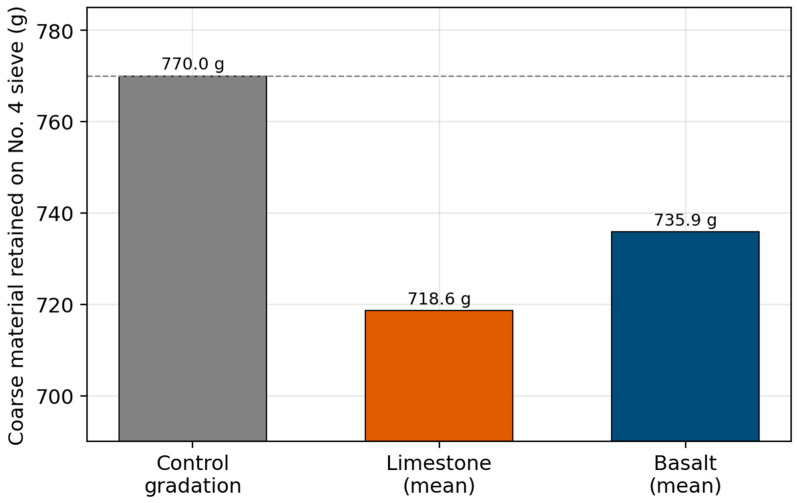
Post-extraction coarse fraction retained on the No. 4 sieve.

Post-extraction gradation analysis was used to examine whether the coarse aggregate fraction changed following Marshall impact compaction (Figure 13). The target gradation contained 770.0 g of material retained on the No. 4 sieve. The corresponding post-extraction group-level values were 718.6 g for the limestone mixtures and 735.9 g for the basalt mixtures. These values were derived from one extracted specimen for each included mixture rather than from replicate extractions of the same mixture condition. Consequently, experimental variability could not be quantified, and no statistical significance can be assigned to the difference between the two aggregate groups.

The lower retained coarse-fraction value observed for the limestone mixtures suggests a possible trend toward greater particle degradation during Marshall compaction. This trend is directionally consistent with the aggregate characterization results, for which limestone exhibited higher Los Angeles abrasion loss, Micro-Deval loss, and flakiness index than basalt (Table 2). Nevertheless, the post-extraction result should be regarded only as a corroborative observation and not as independent proof that basalt preserved the SMA skeleton more effectively. Replicated extraction and sieve analysis would be required to confirm the magnitude and statistical reliability of the lithology-related difference.

**Figure 14:**
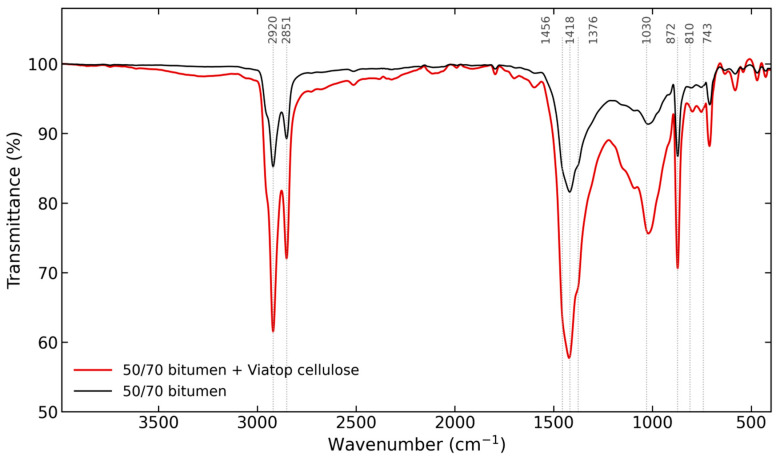
ATR-FTIR transmittance spectra of the neat 50/70 bitumen and the 50/70 bitumen–cellulose fiber combination (dotted lines mark the shared band positions).

The FTIR spectra of the neat 50/70 bitumen and of the bitumen–cellulose fiber combination are compared in Figure 14. Both spectra are dominated by the characteristic absorption bands of the bituminous matrix, as follows: the aliphatic C–H stretching bands at 2920 and 2851 cm^−1^, the C–H bending bands at 1456 and 1376 cm^−1^, the band near 1418 cm^−1^, the S=O/C–O absorption envelope around 1030 cm^−1^, and the out-of-plane aromatic C–H bands at 872, 810 and 743 cm^−1^ [27]. The characteristic bands of cellulose reported in the literature, a broad O–H stretching band near 3340 cm^−1^ and fingerprint bands around 1030 and 898 cm^−1^ [28], were not observed as distinct features in the combination spectrum; only a very weak absorption could be discerned in the 3200–3500 cm^−1^ region. The two spectra share the same band positions, and incorporation of the fiber produced no new absorption bands and no measurable band shifts; the moderate differences in overall band intensity are attributable to differences in ATR contact rather than to a compositional change. These observations indicate that the interaction between the cellulose fiber and the bituminous binder is predominantly physical and that fiber stabilization occurs through binder retention and immobilization of the mastic rather than through chemical modification of the binder [14].

**Figure 15:**
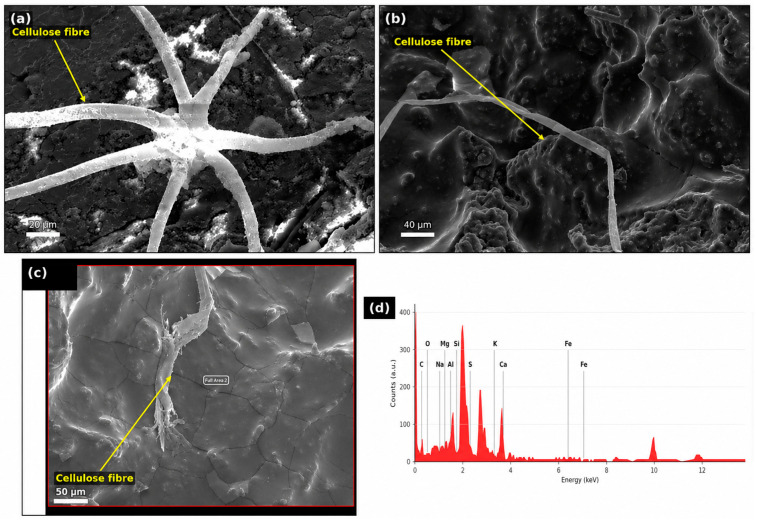
SEM micrographs and EDX spectrum of the cellulose-fiber-stabilized bituminous mastic: (**a**–**c**) fiber morphology and embedment within the mastic, and (**d**) EDX spectrum of a fiber-containing region. In (**a**–**c**), the yellow arrows indicate the cellulose fibre, and the red frame in (**c**) marks the EDX scan area (‘Full Area’). In (**d**), the red curve is the EDX spectrum, with the labeled peaks denoting the detected elements (C, O, Al, Si, Ca, Fe).

SEM micrographs and the accompanying EDX spectrum (Figure 15) further support this interpretation. The images show cellulose fibers embedded within and partially coated by the bituminous mastic, forming a filamentary network that bridges the binder-rich phase rather than dissolving into it. Fiber surfaces remained morphologically distinct, and the surrounding matrix adheres to the fibers, which is consistent with mechanical anchorage and binder holding. The EDX spectrum of a fiber-containing region is dominated by carbon- and oxygen-related signals, together with minor mineral-associated elements from the surrounding mastic/filler phase, confirming the heterogeneous multi-phase character of the system.

It should be emphasized that a physical (rather than chemical) mode of interaction does not imply that the fiber is mechanically inert. The physical mechanisms (free-binder retention, formation of a filamentary fiber network, and the resulting increase in mastic consistency and stiffness) can still modify the mechanical response of the mixture. The FTIR/SEM evidence therefore defines how the fiber acts (physically, without altering the binder chemistry), while the Marshall fiber-content series (Section 3.1) quantifies the magnitude of that physical action; the two observations are complementary rather than contradictory.

When considered together with the Marshall and Schellenberg drainage results, these microstructural observations support a consistent mechanism, as follows: the basalt aggregate skeleton provides the primary load-bearing framework, while the limestone-filler–cellulose-fiber mastic stabilizes the binder-rich phase and limits free-binder movement [10,12]. Thus, the beneficial role of the fiber in this study should be interpreted mainly as physical mastic stabilization rather than chemical modification of the binder. The aggregate-type results should also be considered in relation to the measured difference in water absorption. The basalt aggregate exhibited substantially higher water absorption than limestone, indicating a potentially greater capacity to absorb part of the binder at the aggregate surface. Such absorption could affect the effective binder available within the mastic and the development of the binder film; however, neither binder absorption nor film thickness was quantified in this study. Consequently, the water-absorption difference cannot be used to establish a causal explanation for the volumetric or mechanical differences between the basalt and limestone mixtures. It should instead be regarded as a potentially relevant material characteristic that warrants direct investigation through effective-binder determination and moisture-sensitivity testing.

Limitations and future work. This study was limited to the initial volumetric and Marshall (dry) response of the SMA skeleton–mastic system. Binder absorption was not quantified separately, so the effective binder content and film thickness of the basalt mixtures may be lower than the nominal values; in the conditioning experiment, the thermal exposure and the accompanying densification could not be separated; the post-extraction gradation was not replicated; and the MCDM weights and air-void reference were analyst-defined, with no mixture meeting all design targets simultaneously, so those rankings are relative comparisons rather than compliance statements. Moisture-conditioned and long-term-aged specimens were not produced, so moisture susceptibility and aging- related durability could not be evaluated. Given the higher water absorption of basalt, moisture-sensitivity testing (TSR and Hamburg wheel-tracking) is a particularly relevant next step, and long-term oven aging together with binder aging indices is recommended to assess in-service durability. Performance-based rutting testing and DSR/MSCR performance-grade characterization of the binder are also recommended. These items define a durability-oriented continuation of the present skeleton–mastic evaluation.

## 4. Conclusions

This study evaluated polymer-modified stone mastic asphalt as a skeleton–mastic pavement material by considering aggregate gradation, aggregate type, cellulose-fiber stabilization, binder-content sensitivity, and pre-compaction laboratory conditioning within a single experimental framework. Based on the results obtained, the following conclusions can be drawn:

1. The cellulose-fiber content should be interpreted primarily as a drainage-control design parameter rather than as a purely mechanical reinforcement variable. Increasing the fiber content from 0.30% to 0.35% reduced the mean Schellenberg binder drainage from 0.27% to 0.18% and also reduced the variability of the drainage result. Therefore, 0.35% fiber by total mixture mass was adopted as the drainage-based design fiber content.
2. The preliminary fiber-content series showed that the maximum recorded load within the fixed 10 mm test window remained nearly unchanged between the fiber-free control and the selected 0.35% dosage and increased at the higher fiber contents. However, because the load–deformation curves did not reach a distinct failure peak followed by post-peak softening, the recorded parameters cannot be interpreted as standard Marshall stability, flow, or Marshall quotient. The results therefore provide only a within-series indication of load–deformation sensitivity and do not establish a mechanical optimum. The 1% and 2% fiber contents should be regarded as higher-dosage sensitivity levels, while the selection of 0.35% remains based on drainage control.
3. The response of the SMA mixtures was strongly affected by aggregate gradation and skeleton configuration. The lower- and upper-limit gradations did not show the same volumetric and Marshall response to binder-content variation, indicating that SMA behavior cannot be interpreted from binder content alone. Instead, the aggregate skeleton controls how the mixture responds to changes in binder content and mastic volume.
4. The binder-content series should be considered as a sensitivity analysis rather than a complete optimum binder content determination. Within the investigated binder-content range, the fitted air-void response did not consistently reach the conventional 4% design target, and some response optima occurred near the boundary of the tested range. Therefore, no extrapolated OBC was reported from these data. The MCDM rankings reported here are relative comparisons among the tested alternatives under analyst-defined weights and targets, and are not presented as production optima.
5. The post-extraction gradation results showed an indicative reduction in the coarse fraction retained on the No. 4 sieve, from the target value of 770.0 g to group-level values of 718.6 g for the limestone mixtures and 735.9 g for the basalt mixtures. The lower retained value for limestone was directionally consistent with its higher abrasion losses and flakiness index. However, because replicate extraction testing was not conducted, this difference should be interpreted as a preliminary trend rather than statistically confirmed evidence of superior skeleton preservation by basalt.
6. FTIR analysis of the neat bitumen and the bitumen–fiber combination showed the same band positions, with no new absorption bands and no measurable band shifts after fiber incorporation, indicating that the interaction between the cellulose fiber and the bituminous phase was predominantly physical rather than chemical. SEM observations further supported this interpretation by showing fibers embedded in and partially coated by the bituminous mastic, while EDX confirmed the heterogeneous fiber–binder–filler character of the mastic phase.

Overall, the results support a material-system interpretation of SMA in which the basalt aggregate skeleton provides the main load-bearing framework, while the limestone filler–bitumen–cellulose fiber mastic stabilizes the binder-rich phase and limits free-binder movement. The beneficial role of the cellulose fiber should therefore be interpreted mainly as physical mastic stabilization and binder-retention support rather than chemical modification of the binder. The conditioning results should be interpreted within the limits of the adopted laboratory protocol. Since pre-compaction conditioning was accompanied by changes in air-void content, the observed aggregate-dependent stability trends represent a combined conditioning–densification response rather than a pure aging effect. Further testing at controlled air-void levels is required to confirm the mechanism.

## Figures and Tables

**Table 1:** Physical properties of aggregates.

Property	Standard	Limestone	Basalt
Coarse aggregate volume density (g/cm^3^)	TS EN 1097-6/ASTM C127	2.68	2.56
Coarse aggregate apparent density (g/cm^3^)	TS EN 1097-6/ASTM C127	2.72	2.67
Coarse aggregate absorption (%)	TS EN 1097-6/ASTM C127	0.55	1.61
Fine aggregate volume density (g/cm^3^)	TS EN 1097-6/ASTM C128	2.69	2.57
Fine aggregate apparent density (g/cm^3^)	TS EN 1097-6/ASTM C128	2.72	2.69
Fine aggregate absorption (%)	TS EN 1097-6/ASTM C128	0.41	1.73

**Table 2:** Mechanical properties of aggregates.

Property	Standard	Limestone	Basalt
Los Angeles abrasion resistance (%)	TS EN 1097-2/ASTM C131	27.2	13.7
Micro-Deval abrasion resistance (%)	TS EN 1097-1/ASTM D7428	17.8	10.3
Methylene blue value (g/100 g)	TS EN 933-9/ASTM C1777	6	3
Flakiness index (%)	TS EN 933-3/ASTM D4791	22.8	14.5

**Table 3:** Basic properties of the SBS-modified binder.

Property	Standard	Value
Density (g/cm^3^)	TS EN 15326 + A1/ASTM D70	1.03
Penetration at 25 °C (0.1 mm)	TS EN 1426/ASTM D5	30.5
Softening point (°C)	TS EN 1427/ASTM D36	75.6
Ductility at 25 °C (cm)	TS EN 13589/ASTM D113	100+
Elastic recovery at 25 °C (%)	TS EN 13398/ASTM D6084	83.5
Mass loss after RTFOT, 163 °C (%)	TS EN 12607-1/ASTM D2872	0.14
Post-RTFOT penetration at 25 °C (0.1 mm)	TS EN 1426/ASTM D5	21.4
Post-RTFOT softening point (°C)	TS EN 1427/ASTM D36	79.5

**Table 4:** Properties of the cellulose-fiber additive used in this study.

Property	Value
Trade name	VIATOP^®^ premium
Supplier	J. Rettenmaier & Söhne GmbH + Co. KG, Rosenberg, Germany
Type	Bitumen-coated cellulose-fiber pellets
Pellet diameter (mm)	2–8 mm
Pellet length (mm)	3–5 mm
Bulk density	440–510 g/L
Recommended SMA dosage	0.3 wt% (3.0 kg/ton)
Dosage used in this study	0.35 wt% of mixture
Measured single-fiber diameter (SEM, *n* = 24,252)	mean 9.97 μm; median 6.50 μm

**Table 5:** Measured diameter distribution of the cellulose fibers (SEM image analysis, *n* = 24,252).

Parameter	Value
Number of measured fibers, n	24,252
Mean diameter (μm)	9.97
Median diameter (μm)	6.50
Standard deviation (μm)	13.23
P10–P90 (μm)	1.79–19.57
Minimum–Maximum (μm)	0.63–98.35

**Table 6:** Structure of the experimental series and specimen inventory.

Series	Design Variable	Aggregate	Binder (%)	Fiber (%)	Mixtures/Levels	Spec. (n × 3)
D1	Binder content (lower-limit gradation)	Basalt	5.5/6.0/6.5/7.0	0.35	4 binder levels	12
D2	Binder content (upper-limit gradation)	Basalt	5.5/6.0/6.5/7.0	0.35	4 binder levels	12
D3–D4	Aggregate type and gradation	Basalt/limestone	6.5	0.35	Control basalt; limestone coarse; limestone fine; basalt coarse; basalt fine (5)	15
D5	Aggregate type and pre-compaction conditioning	Basalt/limestone	6.5	0.35	Basalt (uncond./cond.); limestone (uncond./cond.) (4)	12
fiber	Cellulose-fiber content	Basalt (control gradation)	6.5	0/0.35/1/2	4 fiber levels	12
Total	—	—	—	—	—	63

**Table 7:** Schellenberg binder-drainage test results.

Fiber Content (%)	Mean Drainage (%)	SD (%)
0.30	0.27	0.020
0.35	0.18	0.006

**Table 8:** Effect of pre-compaction conditioning on the Marshall response (mean ± SD, *n* = 3). Retained % = conditioned/unconditioned × 100; significance from independent-samples *t*-tests at α = 0.05.

Mixture	Parameter	Unconditioned	Conditioned	Δ %	Retained %	*p* (*t*-Test)	Sig.
Basalt	Stability (kN)	13.86 ± 0.28	12.51 ± 0.51	−9.7	90.3	0.016	Yes
Basalt	Flow (mm)	3.78 ± 0.24	3.50 ± 0.32	−7.4	92.6	0.289	No
Basalt	MQ (kN/mm)	3.67 ± 0.16	3.58 ± 0.18	−2.4	97.6	0.551	No
Basalt	Air voids (%)	6.51 ± 0.50	4.97 ± 0.27	−23.7	76.3	0.009	Yes
Limestone	Stability (kN)	9.27 ± 0.38	10.33 ± 0.41	+11.4	111.4	0.029	Yes
Limestone	Flow (mm)	3.90 ± 0.37	3.67 ± 0.27	−5.9	94.1	0.432	No
Limestone	MQ (kN/mm)	2.38 ± 0.13	2.82 ± 0.10	+18.2	118.2	0.010	Yes
Limestone	Air voids (%)	2.47 ± 0.34	1.82 ± 0.30	−26.3	73.7	0.067	No

## Data Availability

The original contributions presented in this study are included in the article/Supplementary Materials. Further inquiries can be directed to the corresponding author.

## Supplementary Materials

The following supporting information can be downloaded at: https://www.mdpi.com/article/10.3390/polym18141769/s1, Table S1. Mean Marshall and volumetric responses of the aggregate-type mixtures used as the MCDM decision matrix (*n* = 3 per mixture); Table S2. TOPSIS closeness coefficient (C_i_) with and without flow, under equal criterion weights. Ranks are given in parentheses (1 = best).
