# Supplementary material for: Drainage-Controlled Cellulose-Fiber Stabilization and Skeleton–Mastic Response of Polymer-Modified Stone Mastic Asphalt
Source: Polymers (Basel). 2026 Jul 20;18(14):1769. doi: 10.3390/polym18141769 (PMC13419312; doi:10.3390/polym18141769)
Supplement: Supplementary file 1 [file polymers-18-01769-s001.zip › polymers-4443280-supplementary.pdf]

Supplementary Material

Sensitivity of the multi-criteria ranking to the inclusion of flow as a decision criterion

This supplementary analysis quantifies the influence of flow as a multi-criteria decision-making (MCDM) criterion on the ranking of the aggregate-type mixtures (design series D3–D4, 6.5% binder). The analysis was repeated with flow retained (six criteria) and with flow removed (five criteria) using the same decision matrix, normalisation and target definitions described in Section 2.5. To avoid any influence of analyst-defined weighting, equal criterion weights were used as the primary scheme; robustness under an alternative weighting is reported below. Stability, VMA and Marshall quotient (MQ) were treated as larger-the-better criteria, and air voids, VFA and flow as target-the-best criteria with targets of 4%, 70% and 3 mm, respectively.

Table S1. Mean Marshall and volumetric responses of the aggregate-type mixtures used as the MCDM decision matrix (n = 3 per mixture).

Mixture (6.5% binder)	Air voids, Va (%)	VMA (%)	VFA (%)	Stability (kN)	Flow (mm)	MQ (kN/mm)
Control basalt	6.51	18.20	64.25	13.86	3.78	3.67
Limestone-coarse	6.80	18.73	63.68	11.00	2.86	3.85
Limestone-fine	10.24	21.52	52.40	11.39	3.58	3.18
Basalt-coarse	4.51	16.97	73.45	11.04	3.42	3.23
Basalt-fine	4.91	16.76	70.69	12.38	3.91	3.17

MQ = Marshall quotient = stability / flow.

Table S2. TOPSIS closeness coefficient (C_i) with and without flow, under equal criterion weights. Ranks are given in parentheses (1 = best).

Mixture	TOPSIS C_i (rank)	
	With flow (6 criteria)	Without flow (5 criteria)
Control basalt	0.575 (3)	0.605 (3)
Limestone-coarse	0.561 (4)	0.533 (4)
Limestone-fine	0.216 (5)	0.208 (5)
Basalt-coarse	0.686 (1)	0.692 (1)
Basalt-fine	0.647 (2)	0.690 (2)
**Rank correlation vs. with-flow**	**ρ = 1.00, τ = 1.00**	

ρ = Spearman rank correlation; τ = Kendall rank correlation, computed between the with-flow and without-flow scores across the five mixtures.

Robustness and interpretation

Removing flow from the criterion set left the TOPSIS ranking of the five mixtures completely unchanged (ρ = 1.00, τ = 1.00): basalt-coarse remained the highest-ranked alternative and limestone-fine the lowest under equal weights. Under an alternative stability-emphasised weighting scheme (stability weighted three times the other criteria), the TOPSIS ranking was again invariant to the removal of flow (ρ = 1.00), with control basalt ranked highest in both cases.

These results confirm quantitatively that flow contributes negligible independent discriminating information to the multi-criteria evaluation and neither distorts nor dominates the ordering of the tested mixtures. This is consistent with the short loading vector of flow in the PC1–PC2 biplot (Figure 11) and supports the retention of flow among the reported criteria on the grounds of completeness and its role in computing the Marshall quotient, rather than because it influences the ranking.
